# Tumor-intrinsic chromatin programs enforce immune evasion in glioblastoma

**DOI:** 10.1172/JCI203222

**Published:** 2026-03-02

**Authors:** Raymond Sun, Chao Gao, Rongze Olivia Lu

**Affiliations:** 1 Department of Neurological Surgery and; 2 Helen Diller Comprehensive Cancer Center, UCSF, San Francisco, California, USA.; 3 Parker Institute for Cancer Immunotherapy, San Francisco, California, USA.

## Abstract

Immunotherapy has shown limited efficacy in glioblastoma (GBM), reflecting profound immune evasion and an immunosuppressive microenvironment. In this Commentary, we highlight recent work by Zhang and colleagues identifying the transcription factor OLIG2 as a central mediator of immune evasion in GBM. Though OLIG2 has an established role in promoting GBM progression through its effects on glioma stem-like cells (GSCs), Zhang et al. demonstrated a further role for OLIG2 in suppressing antitumor immunity: in human GSCs and GSCs from mouse models of GBM, OLIG2 expression epigenetically repressed the interferon-responsive chemokine CXCL10, thereby limiting cytotoxic T cell infiltration. These findings provide a mechanistic explanation for immune resistance in GBM and support targeting tumor-intrinsic chromatin programs to enhance responses to immunotherapy.

## Glioblastoma is resistant to current immunotherapies

Glioblastoma (GBM) is one of the most lethal human cancers, with immune checkpoint inhibitors providing little clinical benefit (1). This failure has been attributed to profound immune exclusion and a highly immunosuppressive tumor microenvironment in GBMs, yet the tumor-intrinsic mechanisms that actively enforce immune evasion remain incompletely defined (2). While prior work has emphasized mesenchymal transcriptional programs, metabolic stress responses, and myeloid-driven suppression (3), less is known about how tumor cell–intrinsic transcriptional and chromatin states directly shape immune accessibility in GBM.

## OLIG2 suppresses immune recruitment by repressing *CXCL10* transcription

**Figure 1:**
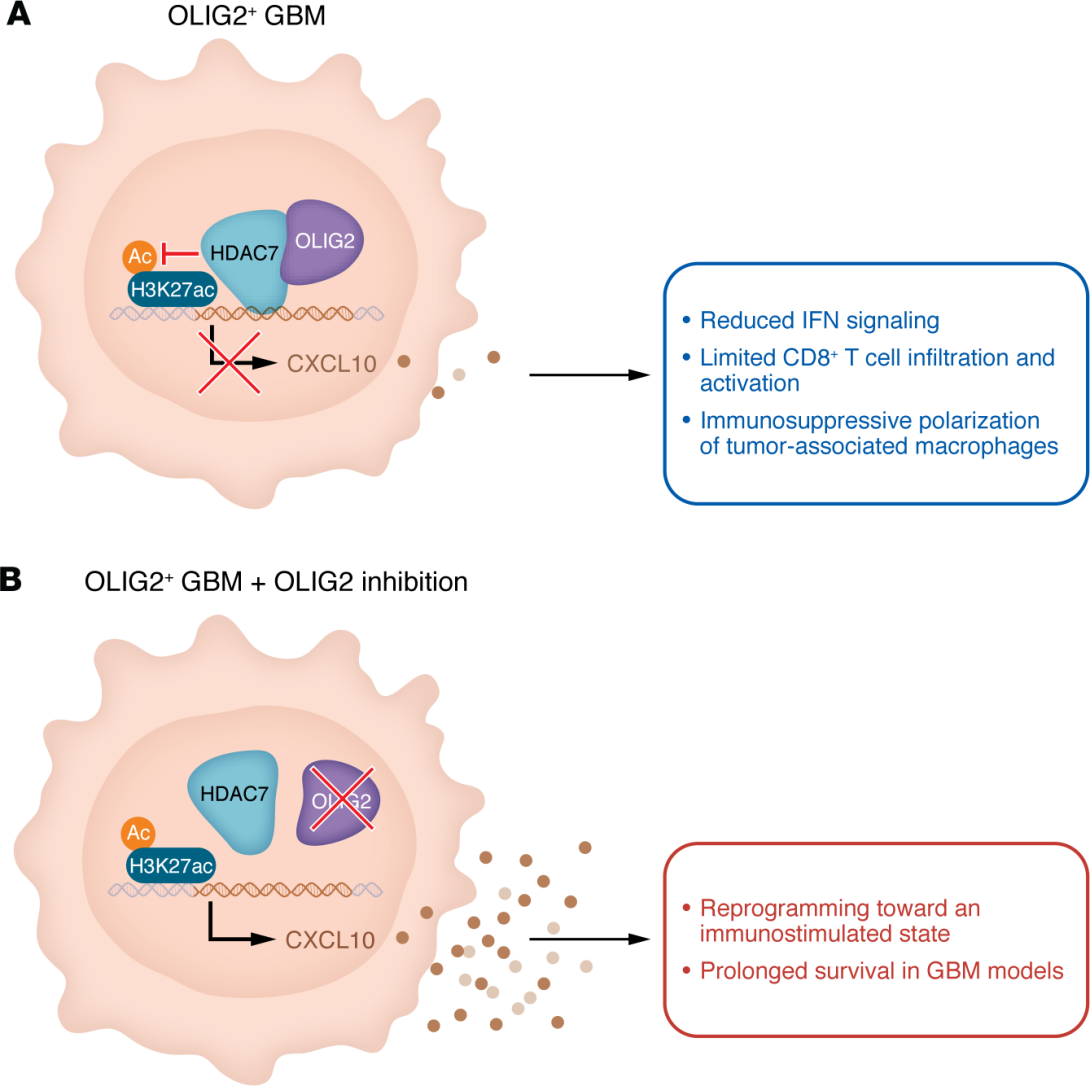
OLIG2 promotes immune suppression in GBM. (**A**) OLIG2 is an oligodendrocyte transcription factor with known roles in tumor proliferation and phenotypic plasticity. Zhang et al. (4) have extended OLIG2’s contribution to GBM by demonstrating its role in limiting antitumor immunity. They reported that OLIG2 recruits HDAC7 to remove H3K27ac enhancers from *CXCL10*. The resulting decrease in CXCL10 signaling led to reductions in IFN signaling and CD8^+^ T cell infiltration as well as immunosuppressive polarization of tumor-associated macrophages, contributing to the “cold” immune phenotype that characterizes GBM. (**B**) In mouse models of GBM, the brain-penetrant OLIG2 inhibitor CT-179 enhanced CD8^+^ T cell activity and sensitized GBM tumors to PD-1/PD-L1 blockade in vivo, prolonging survival. OLIG2 targeting thus represents a potential strategy for reprogramming GBM to overcome immune resistance.

In this issue of the *JCI*, Zhang and colleagues identified the oligodendrocyte lineage transcription factor OLIG2 as a central regulator of immune evasion in GBM (4). OLIG2 is widely expressed in glioma stem-like cells (GSCs) and has been extensively studied for its role in tumor proliferation, lineage maintenance, and phenotypic plasticity (5–7). The work by Zhang et al. substantially extends this paradigm by demonstrating that OLIG2 also actively suppresses antitumor immunity. In investigations incorporating genomic data and immune scores from patients, human GBM cells, and mouse models, Zhang et al. uncovered that, mechanistically, OLIG2 recruited HDAC7 to repress transcription of the chemokine CXCL10, thereby limiting CD8^+^ T cell infiltration and activation while promoting immunosuppressive tumor-associated macrophage polarization. Genetic or pharmacologic inhibition of OLIG2 restored CXCL10 expression, reprogrammed the tumor microenvironment toward an immunostimulatory state, and significantly prolonged survival in multiple GBM models (Figure 1).

OLIG2 is a basic helix–loop–helix transcription factor that regulates oligodendrocyte lineage commitment and maturation during neural development and is broadly expressed in gliomas, where it marks stem-like tumor cell states (5, 8–11). In this article, the findings reposition OLIG2 from a purely tumor-intrinsic driver of growth to a transcriptional gatekeeper that couples glioma lineage identity to immune invisibility (4). Previous models of immune evasion in GBM have largely focused on mesenchymal cell states, inflammatory stress responses, or paracrine signaling from tumor-associated myeloid cells (3, 12). By contrast, this work demonstrates that the OLIG2^+^ stem-like state can actively enforce immune exclusion through direct chromatin-level repression of chemokine signaling. Consistent with this model, Zhang et al. demonstrated that OLIG2^hi^ human GBM samples exhibited reduced interferon signaling and diminished T cell activation, while spatial transcriptomic analyses revealed preferential localization of T cells in OLIG2^lo^ tumor regions (4).

Genetic context further sharpens the model for OLIG2-enforced immune exclusion. OLIG2 is also expressed in isocitrate dehydrogenase–mutant (IDH-mutant) lower-grade glioma, and OLIG2 overexpression is a good surrogate marker for IDH mutation (13, 14). Given the global CpG island hypermethylation characteristic of IDH-mutant gliomas (15), it will be important to determine whether OLIG2 cooperates with IDH-driven epigenetic programs to reinforce silencing of immune regulatory pathways. Such cooperation could further constrain chemokine expression and immune infiltration, helping to explain the immune-excluded phenotype observed even in lower-grade IDH-mutant tumors.

## STING/type I interferon signaling in GBMs

The mechanistic distinction of this pathway is notable. CXCL10 is classically induced downstream of type I interferon and cGAS/STING signaling, a central axis of innate immune sensing that promotes antigen presentation, dendritic cell activation, and cytotoxic T cell recruitment (16–18). However, accumulating evidence indicates that GBM tumors attenuate this pathway through multiple mechanisms, including promoter hypermethylation and transcriptional silencing of interferon-stimulated genes and cGAS/STING components (19). Such epigenetic repression limits chemokine production and contributes to the immune-cold phenotype characteristic of GBMs, even in the presence of inflammatory cues. In contrast to these mechanisms, Zhang et al. showed that OLIG2 suppressed CXCL10 independently of STING/type I interferon activation, acting instead through HDAC7-mediated repression of H3K27 acetylation at CXCL10 regulatory elements (4). These findings suggest that rather than modulating upstream type I interferon signaling, OLIG2^+^ GSCs promote immune evasion by directly repressing the interferon-responsive gene *CXCL10* through epigenetic mechanisms. Whether OLIG2-dependent chromatin repression extends more broadly to interferon-stimulated genes, including components of the STING pathway, remains an important question for future investigation. The present work suggests that effective immunotherapy for GBM may require dismantling tumor-intrinsic chromatin barriers to immune recruitment, rather than solely amplifying upstream interferon signaling.

## Therapeutic implication of targeting OLIG2

The therapeutic implications of this work are compelling. Zhang et al. demonstrated that CT-179, a brain-penetrant OLIG2 inhibitor, enhanced CD8^+^ T cell activity and sensitized GBM tumors to PD-1/PD-L1 blockade in vivo (4). Rather than directly stimulating immune pathways, OLIG2 inhibition dismantled a tumor-intrinsic barrier to immune access, providing a rational strategy to convert immunologically “cold” GBMs into a more proinflammatory state for immunotherapy. This approach complements emerging efforts to activate innate immune sensing and suggests that durable immune engagement in GBMs may require coordinated disruption of multiple tumor-intrinsic immune barriers.

Together, these findings establish OLIG2 as a key molecular regulator of immune evasion in GBM and highlight chromatin-based mechanisms as a promising avenue for overcoming immune resistance.

## Funding support

This work is the result of NIH funding, in whole or in part, and is subject to the NIH Public Access Policy. Through acceptance of this federal funding, the NIH has been given a right to make the work publicly available in PubMed Central.

- NIH grant R01NS126501 (to ROL).

## Version 1 03/02/2026

Electronic publication
